# Application of neural networks with back-propagation to genome-enabled prediction of complex traits in Holstein-Friesian and German Fleckvieh cattle

**DOI:** 10.1186/s12711-015-0097-5

**Published:** 2015-03-31

**Authors:** Anita Ehret, David Hochstuhl, Daniel Gianola, Georg Thaller

**Affiliations:** Institute of Animal Breeding and Husbandry, Christian-Albrechts-University Kiel, Olshausenstr. 40, Kiel, 24098 Germany; Institute of Theoretical Physics and Astrophysics, Christian-Albrechts-University Kiel, Leibnizstr. 15, Kiel, 24098 Germany; Department of Animal Sciences, University of Wisconsin-Madison, Madison, 53706 USA; Department of Dairy Science, University of Wisconsin, Madison, 53706 USA; Department of Biostatistics and Medical Informatics, University of Wisconsin, Madison, 53706 USA

## Abstract

**Background:**

Recently, artificial neural networks (ANN) have been proposed as promising machines for marker-based genomic predictions of complex traits in animal and plant breeding. ANN are universal approximators of complex functions, that can capture cryptic relationships between SNPs (single nucleotide polymorphisms) and phenotypic values without the need of explicitly defining a genetic model. This concept is attractive for high-dimensional and noisy data, especially when the genetic architecture of the trait is unknown. However, the properties of ANN for the prediction of future outcomes of genomic selection using real data are not well characterized and, due to high computational costs, using whole-genome marker sets is difficult. We examined different non-linear network architectures, as well as several genomic covariate structures as network inputs in order to assess their ability to predict milk traits in three dairy cattle data sets using large-scale SNP data. For training, a regularized back propagation algorithm was used. The average correlation between the observed and predicted phenotypes in a 20 times 5-fold cross-validation was used to assess predictive ability. A linear network model served as benchmark.

**Results:**

Predictive abilities of different ANN models varied markedly, whereas differences between data sets were small. Dimension reduction methods enhanced prediction performance in all data sets, while at the same time computational cost decreased. For the Holstein-Friesian bull data set, an ANN with 10 neurons in the hidden layer achieved a predictive correlation of *r*=0.47 for milk yield when the entire marker matrix was used. Predictive ability increased when the genomic relationship matrix (*r*=0.64) was used as input and was best (*r*=0.67) when principal component scores of the marker genotypes were used. Similar results were found for the other traits in all data sets.

**Conclusion:**

Artificial neural networks are powerful machines for non-linear genome-enabled predictions in animal breeding. However, to produce stable and high-quality outputs, variable selection methods are highly recommended, when the number of markers vastly exceeds sample size.

## Background

In genome-enabled prediction of traits in animal and plant breeding, building appropriate models can be extremely challenging, especially when the association between predictors and target variable involves non-additive effects [1-4]. Linear methods that are frequently used in genome-enabled predictions typically ignore gene by gene interactions, as well as higher order non-linearities. To meet this challenge, and to take possible non-linearities into account in prediction, there has been a growing interest in the use of semi- and non-parametric methods [3,5,6]. In this context, machine learning methods and, in particular, artificial neural networks (ANN) have been considered to be promising predictive machineries [7-9].

Nevertheless, there have been only a few empirical applications of ANN to genome-enabled prediction in animal and plant breeding. In the next paragraphs, we will first give a short overview of this method and of the state of the art in the field of animal breeding.

**Figure 1:**
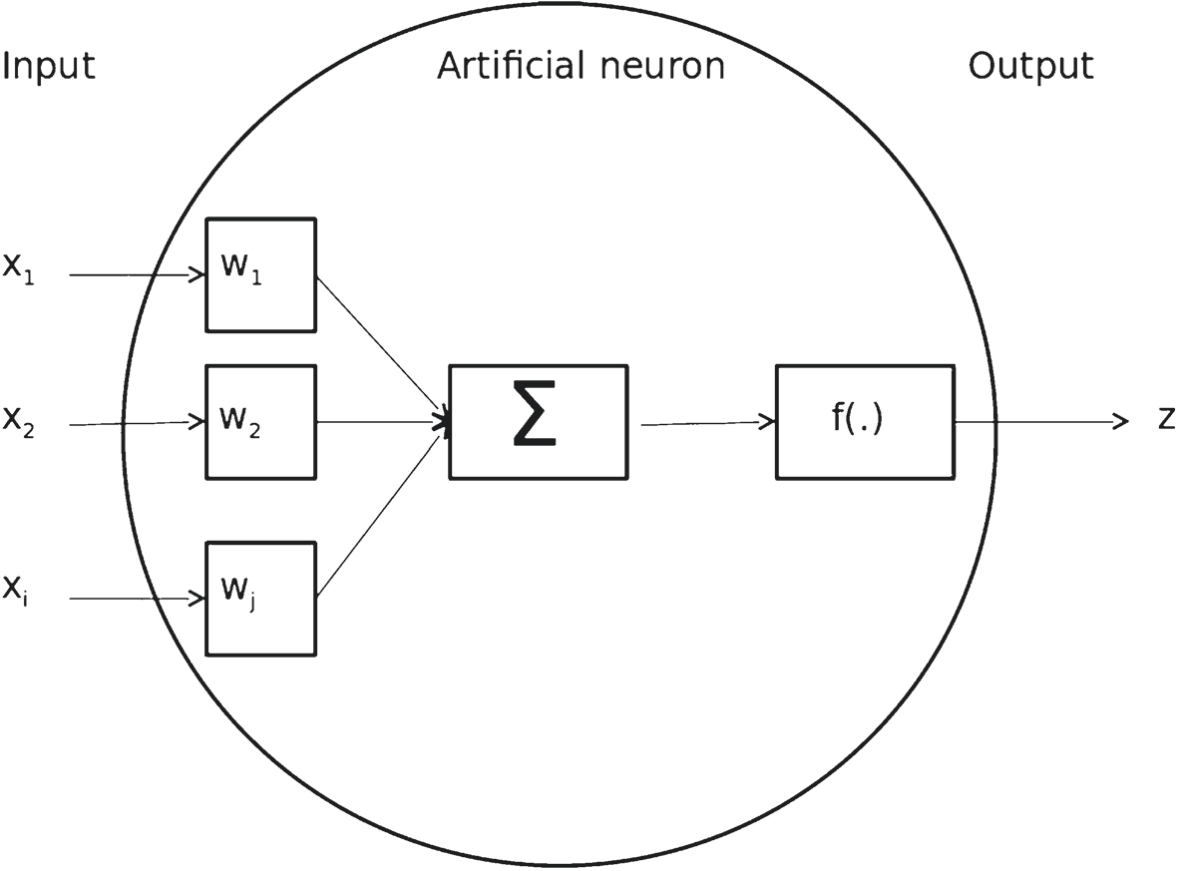
**Schematic representation of an artificial neuron.** *x*_*i*_ = input value; *w*_*j*_ = weights linked to single input values; *f*(.) = activation function of the artificial neurons; *z* = output of artificial neuron; $\sum $ indicates some computation.

Initially ANN were developed in the field of artificial intelligence and were first introduced for image recognition. The central concept was inspired by knowledge of the nervous system, especially the human brain with its closely connected neurons [10]. The idea has been used to define statistical models in the form of neuron diagrams, as shown in Figure 1. In an idealized artificial neuron, all received input information *x*_*i*_ (*i*= number of inputs, e.g., marker genotypes) is weighted via appropriate elements *w*_*j*_ and summed up. The sum of all weighted inputs is transformed by an activation function *f*(.) to produce the neuron output *z* (e.g., the predicted phenotype). The activation function can be either linear or non-linear and its purpose is to restrict the amplitude of the neuron’s output.

To mimic the physical structure of the human nervous system, artificial neurons are interconnected and organized in networks with several layers, which together form the ANN. In most applications, the information usually flows straight forwardly, thus the output of one neuron forms the input of another one. Algebraically, an ANN can be represented as a schematic of Kolmogorov’s theorem [11] for the representation of complex functions, by which ANN are proven to be universal function approximators [12]. Therefore, in the context of genome-enabled prediction [13], ANN are theoretically able to account for cryptic interactions between genotypes and phenotypes without the need of explicitly specifying a fixed genetic model [7], thus the genetic architecture of the trait can remain unknown a priori.

In short, an appealing property of ANN is that they do not require most of the prior assumptions that commonly underlie parametric statistical models. ANN can be non-linear in both features and parameters and, if properly specified, may capture complex signals from the data and deliver a better predictive accuracy. This is achieved through a learning phase where, for example, several pairs of genotype-phenotype combinations are fed into the network. According to a specific learning rule, the ANN can memorize the function of training samples. Learning is an iterative process, where at each iteration the weights (connections between single artificial neurons) of the ANN are steadily adjusted, in order to minimize the difference between observed and predicted output (e.g., phenotypes) or training error [14]. The process is stopped when a previously defined threshold of the error function in training samples is reached. Hence, adjusting the weights properly to a given mapping problem is an optimization task. The most widespread supervised learning rule for ANN is the back-propagation of error algorithm [15]. It is a supervised learning algorithm and can be seen either as a gradient descent method to locate the optimal solution [16], or as a generalization of the delta rule [17]. The algorithm is based on minimization of the error function with respect to the weights and in general applies a least-squares solution, so that the procedure can be viewed as a non-linear regression algorithm. A trained ANN is able to predict unknown future outcomes of the same physical process that created the training samples.

ANN are flexible and powerful and can be implemented in various ways [5]. In animal breeding, ANN were claimed to have the ability of outperforming frequently used standard linear regression models in prediction of yet to be observed phenotypic values through genomic data [7-9]. However, in the context of genome-enabled prediction they are pretty good predictive machines but through their so called black-box-behaviour they cannot be used to make any inference of SNPs (single nucleotide polymorphisms)on phenotypes. ANN are computationally costly, especially when applied to high-dimensional genomic data, for which the number of parameters to be estimated typically exceeds the number of available samples. Therefore, at least in animal breeding, only subsets of markers have been used to make ANN computational feasible [9,18].

This study is the first application that uses an entire genomic marker set as source of input information for genome-enabled prediction of milk traits in dairy cattle. The predictive performance of ANN will depend on network architecture, training phase and the characteristics of the data [7]. In order to assess the importance of these factors, we applied different ANN structures to prediction of yet to be observed phenotypic values from large-scale SNP data. A linear ANN with one neuron in the hidden layer and with the genomic relationship matrix [19] used as input information served as a benchmark. Such an ANN produces results approximately corresponding to those of GBLUP (genomic best linear unbiased prediction) [7,20], which is a standard method for genome-enabled prediction in animal breeding.

## Methods

### Single hidden layer feed-forward ANN with back-propagation

**Figure 2:**
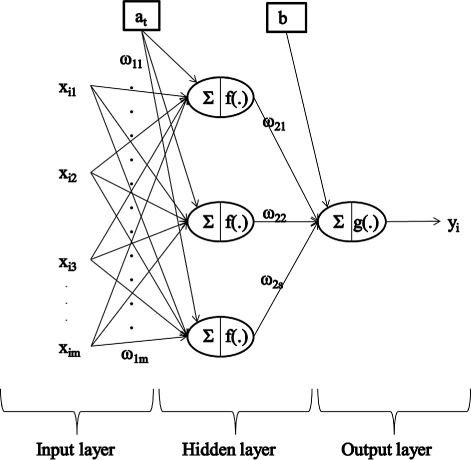
**Architecture of a two-layer feed forward neural network.** *x*_*ij*_ = network input, e.g., marker genotype *j* of individual *i*; *w*_1*m*_ = network weight from the input to hidden layer; *w*_2*s*_ = network weight from the hidden to the output layer; *y*_*i*_ network output, e.g., predicted phenotype of individual; *f*(.) = activation function at the hidden neurons; *g*(.) = activation function at the output neuron; $\sum $ indicates some computation.

Research on theory and applications of artificial networks is steadily growing. Several types of ANN have been extensively used for various purposes, such as classification, pattern recognition, prediction and forecasting, process control, optimization and decision support [21]. A frequently used type of ANN for regression and forecasting is the two-layer feed-forward perceptron [22], also called single hidden layer feed-forward neural network. These are ANN for which an input layer of source nodes and an output unit are completely linked, with only one hidden layer between them, as illustrated in Figure 2. This kind of network can reproduce most mathematical functions fairly well, while keeping a simple architecture. Moreover, it has good properties when working with high-dimensional data, as it is the case in genome-enabled predictions [7].

Mathematically, the mapping of such an ANN can be viewed as a two-step regression [22]. The central concept is to extract in the hidden layer linear combinations of the inputs as basis functions and then model the target as a function of these basis functions in the output layer. In terms of genome-enabled prediction, in the hidden layer, the genomic covariates *x*_*ij*_ (for *j*=1,…,*m*, where *m* denotes the number of genomic covariates) of an individual *i* (for *i*=1,…,*n*) are linearly combined with a vector of weights $w_{1j}^{[t]}$ that are specified in the training phase, plus an intercept (in ANN’s terminology also called “bias”) *a*_*t*_ with *t*=1,…,*s* denoting a neuron. The resulting linear score is then transformed using an activation function *f*_*t*_(.) to produce the output of the single hidden neuron

(1) $$ z_{i}^{[t]} \ = \ f_{t} \left(a_{t} + \sum_{j=1}^{m} w_{1j}^{[t]} x_{ij}\right).  $$

In order to model non-linear relationship between phenotype and input, the hyperbolic tangent activation function ($\tanh (x) = \frac {e^{x} - e^{-x}}{e^{x} + e^{-x}}$) can be used in the hidden neurons, giving the ANN a greater flexibility than that of standard linear regression models [23].

In the output layer, the *s* genotype-derived basis functions, resulting from the hidden layer, are also linearly combined by using the *w*_21_,*w*_22_,…,*w*_2*s*_ weights and an output intercept *b*. In the output neuron, the resulting linear score is transformed, this time by a linear activation function *g*_*t*_(.), to calculate the predicted phenotype of individual *i* (for *i*=1,…,*n*), as

(2) $$ y_{i} \ = \ g_{t} \left(b + \sum_{t=1}^{s} w_{2t} z_{i}^{[t]} \right).  $$

The linear activation function is often an identity function.

At the training phase, the ANN locates the optimal weights by minimization of an error function of the training set. Here, a back-propagation algorithm was used for training. It is a convenient and simple iterative algorithm that usually performs well, even with complex data. Unlike other learning algorithms (like Bayesian learning) it has good computational properties when dealing with large-scale data. To enforce the network generalization ability, regularization was used for training, since this is not naturally achieved through the learning algorithm. The algorithm either was stopped when the maximum number of 1000 iterations was reached (early stopping; [24]), or when the averaged mean squared error (*aMSE*) between predicted and true phenotype reached a certain threshold (*aMSE*≤10^−3^) (phenotypic values were normalized to approximately [−1,1]). Parameters of the learning algorithm were optimally adjusted in the individual data sets in a pre-processing step, and subsequently were held constant for all following runs. In each training round, the weights were initialized to small random values (ranging between −0.1 and 0.1). This helps the algorithm to find the optimal solution. Beyond choosing the best training configuration of the learning algorithm, we examined different network architectures to assess the best predictive ANN. Up to 20 neurons in the hidden layer were tested for their influence on predictive quality. All ANN calculations were performed using a C++ program (written by the authors and available upon request) while pre-processing of the data was done with the publicly available statistical software R [25].

### Benchmark model

To compare the non-linear ANN models with a standard method used in animal breeding, all data sets were also evaluated using a quasi GBLUP. Here, the input variable was the genomic relationship matrix (**G**), which was fed into an ANN with one neuron in the hidden layer and linear activation functions in the hidden as well as in the output layer. The network is similar to GBLUP, in the sense that the network performes a multiple linear regression, in which the weights of the hidden layer can be interpreted as regression coefficients. When **G** is proportional to **XX**^*T*^, where **X** is the incidence matrix of a linear regression model on markers, this is equivalent to ridge regression [7,20], as it is the case in GBLUP [26].

### Phenotype and genotype data

To evaluate the impact of data structure, three data sets were presented separately to the ANN. Inputs were genomic data on 3 341 German Fleckvieh bulls, 2 303 Holstein-Friesian bulls, and 777 Holstein-Friesian dams. All animals were genotyped with a 50k SNP-panel and, after quality control, 39 344, 41 995 and 41 718 SNP markers were used in the analyses respectively, as shown in Table 1. Quality control included eliminating SNPs with a minor allele frequency <0.05 and missing genotype frequency >0.95. For the remaining loci, missing genotypes were imputed using the population-based imputing algorithm Minimac [27], a computationally efficient extension of MaCH which takes pre-phased haplotypes as inputs [28].

**Table 1:** **Data used**

	**Animals in analysis**	**Number of markers after**	**Type of phenotype**
		**quality control**	**records**
German Fleckvieh bulls	3 341	39 344 SNPs	DYD of milk traits
Holstein-Friesian bulls	2 303	41 995 SNPs	DYD of milk traits
Holstein-Friesian cows	777	41 718 SNPs	YD of milk traits

SNP = single nucleotide polymorphism, YD = yield deviations, DYD = daughter yield deviations.

We used three milk traits i.e., milk, fat and protein yield. For the Holstein-Friesian and German Fleckvieh bulls, daughter yield deviations (DYD) were used as phenotypes and, for the Holstein-Friesian cows, yield deviation (YD) was the response variable. A summary of the phenotypes is in Table 2.

**Table 2:** **Summary statistics of phenotypes used**

	**Mean**	**Variance**	**Min**	**Max**
*German Fleckvieh bulls*				
Milk yield DYD	1 779.16	219 257.40	-852.48	3 372.67
Protein yield DYD	59.34	214.18	-23.56	108.65
Fat yield DYD	59.34	320.81	-39.12	137.11
*Holstein-Friesian bulls*				
Milk yield DYD	707.44	434 324.64	-852.09	3 706.01
Protein yield DYD	41.88	391.42	-24.19	104.57
Fat yield DYD	41.14	645.42	-45.81	139.74
*Holstein-Friesian cows*				
Milk yield YD	3.26	26.13	-14.36	19.37
Protein yield YD	0.91	1.54	-4.86	4.23
Fat yield YD	0.21	0.50	-4.17	1.80

DYD = daughter yield deviations, YD = yield deviations.

Feature scaling was applied to the data sets, to enhance numerical stability. This is needed, since otherwise the learning algorithm may not work properly [22]. Feature scaling ensures that all sources of information are treated equally in the training process since it often has a large influence on the final solution. In particular, inputs and outputs must be in the same scale, i.e., ranging approximately from −1 to 1. For all phenotypes the following normalization was used

(3) $$ y_{i}^{\ast} \ = \ \frac{y_{i}-\mu_{y}}{\max_{y}},  $$

where *μ*_*y*_ is the sample mean of the variable and max*y* is its maximum.

### Genomic information

To test the impact of different genomic inputs on the ability of the ANN to predict yet to be observed phenotypes, three genomic covariate structures were used for all data sets. First, the raw genomic marker matrix **X**={*x*_*ij*_} of all SNPs of all individuals was used. **X** is of dimension *n*×*m*, where *n* is the number of animals and *m* the number of markers. Here, feature scaling was done by coding SNP genotypes as −1, 0, and 1 for the homozygote for the minor allele, heterozygote, and homozygote for the other allele, assuming additive allele effects. Second, towards the aim of reducing model complexity and computational cost, genome-derived relationships among individuals were also used as inputs. The genomic relationship matrix **G**={*g*_*ij*_} was calculated following [19]

(4) $$ \mathbf{G} \ = \ \frac{\mathbf{X} \mathbf{X}^{T}}{2\sum_{j=1}^{m} q_{j} \left(1-q_{j}\right)}.  $$

Here, **G** is a standardized genomic relationship matrix, where genotype codes in **X**={*x*_*ij*_} are centred by subtracting their expected frequencies (*q*_*j*_) at each locus. The dimension of the resulting matrix is *n*×*n*. Third, to minimize the loss of information in the original marker matrix, while keeping the dimension small, principal component scores (**UD**) of **X** were used as inputs as well. The **UD** is obtained from the singular value decomposition of **X** [29],

(5) $$ \mathbf{X} \ = \ \mathbf{U} \mathbf{D} \mathbf{V}^{T}.  $$

Here, **U** is an *n*×*n* orthogonal matrix, where the columns consists of eigenvectors of **XX**^*T*^. **D** is a matrix of dimension *n*×*m* containing the square roots of the non-zero eigenvalues of **XX**^*T*^ on the diagonal, and columns of the *m*×*m* matrix **V** are the eigenvectors of **X**^*T*^**X** [8].

For feature scaling, the **G** and **UD** matrices were linearly transformed using the normalization function of the package *brnn* [20] in the R program [25], so all elements of the resulting input matrices ranged between −1 and 1.

### Model validation

To compare the predictive abilities of the ANN models in the different scenarios, a five-fold cross validation scheme was applied and repeated 20 times [30]. The data sets were randomly divided into five subsets of genotypes and associated phenotypes, thus the folds contain different age structures of animals. One subset (testing set) was omitted to test the predictive ability of the model, whereas the other four subsets were used as training samples (training set) to estimate model parameters. During cross-validation runs, each of the five generated subsets served as testing set in one round, with missing phenotypes. At each round, Pearson’s correlation coefficient (*r*) between observed and predicted phenotypes in the testing set was calculated. Since 20 different randomizations were used to assign the genotype-phenotype combinations to five folds, this scheme yielded 100 independent cross-validation runs. Across the single runs, different initializations of weights of the back-propagation algorithm in the range of [−0.1,0.1] were used. This procedure was used to avoid the algorithm repeatedly getting stuck in a local minimum of the error function. The predictive ability of each model reported here was Pearson’s correlation between observed and predicted phenotype values averaged over all 100 individual cross-validation runs.

## Results and discussion

**Figure 3:**
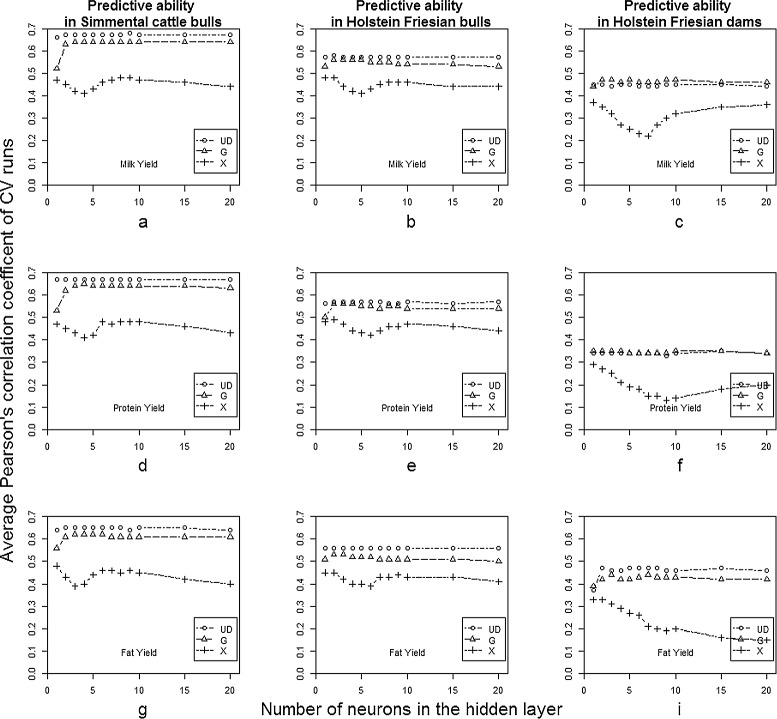
**Comparison of predictive abilities for all scenarios.** Different data sets are in the columns, in rows milk, protein and fat yield are shown. Panels **(a-h)** show the average Pearson’s correlation coefficients over cross-validation runs on the vertical axis, and the number of hidden neurons tested on the horizontal axis. Results of different genomic covariate structures used as inputs (**X**, **G**, **U** **D**) are presented in each panel.

Figure 3 presents the combined results for all prediction scenarios tested. It includes results from different data sets in columns, with milk yield, protein yield and fat yield as response variable in the rows. The single panels (*a*−*h*) show the dependency of the average Pearson’s correlation coefficients of cross-validation runs on network architecture (1 to 20 neurons in the hidden layer) for different genomic covariate structures (**X**, **G**, **UD**) used as input to the ANN.

### Predictive ability of different ANN architectures and input factors

The ANN models differed little in predictive performance in terms of number of neurons in the hidden layer when either the **G** matrix or the **UD** matrix was used. This was observed in all data sets for all traits, as shown in Figure 3. Results are consistent with those of [18], who showed that predictive ability of ANN models did not depend on network architecture when sample size was larger than the number of markers used in the analyses. Our results agreed when the number of features was equal to sample size (**G** and **UD**). Results obtained using the **G** matrix as input to the network are consistent with those of [7], who used Bayesian regularized artificial networks for genome-enabled predictions of milk traits in Jersey cows. Even with much larger data sets and a different training algorithm, we also found that increasing the number of neurons up to 6 neurons yielded slightly better predictions of yet-to-be-observed values, than when using very simple architectures (1 or 2 neurons in the hidden layer) when the **G** matrix is used as input to the network.

Furthermore, predictive abilities of the ANN models in the bull data sets were slightly better when using the **UD** matrix than when using **G** as inputs to the network. Differences between these two inputs in the cow data set were negligible. The same result was obtained by [8] when radial basis function networks were used to predict litter size in pigs. This might be due to the fact that the **UD** decomposition re-expresses the data, taking its variance into account. This mapping may be more accurate and numerically stable for predictions than using **G**.

However, when the marker matrix **X** was fed into the ANN, so the number of features greatly exceeded sample size, Pearson’s correlation coefficients in the cross-validation runs depended tightly on the number of neurons used in the hidden layer of the network. A similar pattern was obtained in all data sets and traits. Averaged over all three data sets, the correlations achieved differed by 0.09 across different network architectures, when the **X** matrix was used. The maximum range was achieved with the Holstein-Friesian dams for protein yield (Figure *3**h*) for which *r* ranged from 0.13 to 0.29 across different architectures. The minimum range of 0.07 (r= [0.41,0.48]) across different numbers of neurons in the hidden layer of the ANN was obtained with the German Fleckvieh data set for milk and protein yield (Figure *3**d*, *3**g*).

The results indicate, that when using **X**, so that the number of features is larger than sample size, a relatively simple ANN architecture (2−6 neurons in the hidden layer) is not able to learn specifications of the data, whereas with complex architectures (over 15 neurons in the hidden layer), the ANN will learn irrelevant details of the data. These are phenomena called under- and over-fitting, respectively. Both types of architectures make prediction of future target values worse. In such situations, less parameterized models, such as linear models, might be advantageous. Furthermore, prediction performance was substantially worse when using the whole marker set as input into the network. This is probably due to over-parameterization of the model, since the number of effective parameters increases quickly with the number of inputs. The effect was independent of the data set used, and using the **X** matrix increased computational cost markedly. Required memory and run times scale approximately as $\mathcal {O}(N_{\textit {markers}} \times N_{hidden units})$. In practice, this amounts to roughly 6 MB of memory for the largest networks used in this work (about 40 000 markers and 20 hidden neurons) and runtime of about one minute for a single iteration. Runtime is ten times greater with the **X** matrix than with the **G** or **UD** matrices used as input to the network.

This results in a ten time increase of runtime when the **X** matrix in comparison to **G** or **UD** was used as input to the networks.

When using the Holstein-Friesian dams data set, the pattern described above was slightly different, notably when protein and fat yields were predicted (Figure 3*f*,3*i*). This might be due to the low sample size compared to marker data in this data set (over-parameterization of ANN models). Furthermore, in all runs learning was stopped when the maximum number of iterations was reached, instead of reaching the optimal *aMSE* in the training configuration.

Moreover, when the **X** matrix was used, even an architecture with one neuron in the hidden layer led to good predictions, since it corresponds approximately to a multiple linear regression on marker genotypes. These results confirm the strength of linear methods in *p*>>*n* problems.

### Predictive ability across different data sets and traits

The highest average correlations between observed and predicted phenotypes in the testing sets were obtained with the German Fleckvieh data set, followed by the prediction of DYD in the Holstein-Friesian bull data set. Prediction of future YD in Holstein-Friesian dams was the worst. Using YD yielded lower correlation coefficients and the ANN failed to give accurate predictions of measured YD values. This is simply because DYD are always based on much more information than YD. However, this might be also influenced by the lower number of animals in the cow data set.

Within data sets, the predictive ability of the models varied only slightly between traits (Figure 3). This might reflect strong additive action for the traits examined, and the fact that the linear pre-corrections used for processing the phenotype tend to normalize the distributions. Thus, the traits behaved similarly, as expected, because phenotypic and marker-based genetic correlations between traits were high, as shown in Table 3.

**Table 3:** **Phenotypic and marker-based genetic correlations between traits within data sets**

***German Fleckvieh bulls***			
	Milk yield DYD	Protein yield DYD	Fat yield DYD
Milk yield DYD	0.87(0.04)	0.58(0.03)	0.73(0.04)
Protein yield DYD	0.70(0.01)	0.79(0.04)	0.62(0.03)
Fat yield DYD	0.89(0.01)	0.81(0.01)	0.77(0.04)
***Holstein-Friesian bulls***			
	Milk yield DYD	Protein yield DYD	Fat yield DYD
Milk yield DYD	0.67(0.05)	0.24(0.04)	0.52(0.04)
Protein yield DYD	0.43(0.02)	0.82(0.05)	0.42(0.04)
Fat yield DYD	0.86(0.01)	0.63(0.01)	0.60(0.04)
***Holstein-Friesian cows***			
	Milk yield YD	Protein yield YD	Fat yield YD
Milk yield YD	0.61(0.08)	0.24(0.06)	0.51(0.08)
Protein yield YD	0.48(0.03)	0.67(0.08)	0.31(0.07)
Fat yield YD	0.92(0.01)	0.60(0.02)	0.51(0.08)

On diagonal of singular panels the marker-based heritability is shown, on the upper off-diagonal the marker-based genetic correlation and on the lower off-diagonal the phenotypic correlation are presented, Standard errors (SE) are shown in brackets, DYD = Daughter yield deviation, YD = Yield deviation.

### Predictive ability compared to a standard linear model

In addition, we investigated a linear ANN, which performs as a quasi GBLUP, as a benchmark model. As shown in Table 4, more complex non-linear architectures could not outperform the linear ANN, i.e. a multiple linear regression on marker relationships. Differences between linear ANN and best predictive ANN were very small. Nevertheless, the linear ANN was superior to a non-linear ANN with the same architecture, although they were fed with the same input information (**G** matrix). This pattern was consistent over all data sets, independently of the trait investigated, and pronounced when no dimension reduction of input to the network was made. Overall, the results indicate that linear methods are reliable when working with large-scaled data, and provide results that are as good as the much more computationally intensive non-linear ANN when milk traits are used as response variable in genome-enabled prediction.

**Table 4:** **Model comparison of linear and non-linear ANN models**

	**Linear ANN**	**Non linear ANN**	**Best non-linear ANN**
	**r**	**r**	**r**
*German Fleckvieh bulls*			
Milk yield DYD	0.68 (0.0007)	0.52 (0.0016)	0.68 (0.0008)
Protein yield DYD	0.68 (0.0006)	0.53 (0.0011)	0.67 (0.0005)
Fat yield DYD	0.66 (0.0005)	0.56 (0.0008)	0.65 (0.0005)
*Holstein-Friesian bulls*			
Milk yield DYD	0.60 (0.0006)	0.53 (0.0011)	0.58 (0.0008)
Protein yield DYD	0.59 (0.0009)	0.50 (0.0013)	0.57 (0.0009)
Fat yield DYD	0.57 (0.0009)	0.51 (0.0010)	0.56 (0.0009)
*Holstein-Friesian cows*			
Milk yield YD	0.47 (0.0031)	0.44 (0.0040)	0.47 (0.0027)
Protein yield YD	0.37 (0.0033)	0.35 (0.0039)	0.35 (0.0032)
Fat yield YD	0.46 (0.0037)	0.39 (0.0049)	0.47 (0.0028)

Compared are linear and non-linear ANN with 1 neuron in hidden layer and **G** matrix as input to the network and best non-linear ANN. DYD = Daughter yield deviation, YD = Yield deviation, r = average Pearson correlation coefficient of the cross-validation runs, variance of cross-validation runs is shown in brackets.

## Conclusions

We used several ANN models for genome-enabled prediction using large-scale SNP-panels and investigated the influence of various inputs and architectures with milk traits in different dairy cattle data sets. The aim was to assess the impact of data structure, type of genomic information used as input to the network, and network architecture on the predictive performance.

Our results indicate that dimension reduction yields higher, more accurate and more consistent predictions of future phenotypes, irrespective of trait and data set used. Thus, we recommend feature selection methods and regularization in the training phase of an ANN (e.g., weight decay [17]) for genome-enabled predictions on large SNP-panels. In this context, the large number of parameters in a richly structured ANN impairs its predictive power, and our results confirm the robustness of linear methods. However, we wish to underline the potential of ANN for mapping non-linear relationships between genotype and phenotype. Perhaps ANN maybe more useful for functional traits (or traits were e.g., epistasis is present) than for milk traits, which seem to behave additively and can be predicted well with linear methods. Nevertheless, back-propagation with early stopping [24] is a useful learning algorithm for ANN for genome-enabled predictions from large-scale SNP information, in the sense that a regularized back-propagation learning algorithm keeps computational cost as low as possible, while maintaining good predictive performance, when feature selection is used.
